# Incidental Hepatic Findings in Cardiac Magnetic Resonance Imaging Examinations in Patients with Congenital Heart Disease: A Pilot Study †

**DOI:** 10.3390/jcm15062453

**Published:** 2026-03-23

**Authors:** Gretha Hecke, Bianca Haase, Nikolaus Clodi, Karolin Hauptvogel, David Plajer, Jakob Spogis, Anja Hanser, Jürgen F. Schäfer, Konstantin Nikolaou, Johannes Nordmeyer, Sarah Nordmeyer

**Affiliations:** 1 Faculty of Medicine, Campus Salzburg, Paracelsus Medical University, 5020 Salzburg, Austria; n.clodi@salk.at; 2 Department of Pediatrics, Children’s Hospital of Kreiskliniken Reutlingen, 72764 Reutlingen, Germany; bianca.haase@kliniken-rt.de; 3 Faculty of Medicine, Campus Nuernberg, Paracelsus Medical University, 90419 Nuernberg, Germany; 4 Department of Diagnostic and Interventional Radiology, University Hospital Tuebingen, 72076 Tuebingen, Germany; david.plajer@med.uni-tuebingen.de (D.P.); jakob.spogis@med.uni-tuebingen.de (J.S.); anja.hanser@med.uni-tuebingen.de (A.H.); juergen.schaefer@med.uni-tuebingen.de (J.F.S.); konstantin.nikolaou@med.uni-tuebingen.de (K.N.); sarah.nordmeyer@med.uni-tuebingen.de (S.N.); 5 Department of Paediatric Cardiology and Intensive Medicine, University Hospital Tuebingen, 72076 Tuebingen, Germany; johannes.nordmeyer@med.uni-tuebingen.de

**Keywords:** liver lesion, CHD, cMRI, Fontan, Glenn, TGA

## Abstract

**Objectives**: During cardiac magnetic resonance imaging (cMRI) exams in patients with congenital heart disease (CHD), incidental liver abnormalities are increasingly found. However, no systematic data exist on the incidence of liver lesions in patients with different CHDs. In order to gain a first overview, we retrospectively analyzed cMRI examinations from the last 10 years at our institution. **Methods**: CMRI examinations including T2-weighted images covering parts of the liver were performed on 899 patients with CHD at our institution between 2014 and 2024. The cMRI examinations were analyzed by a medical student, a pediatrician, a radiologist, and a pediatric cardiologist. Liver lesions were defined as atypical liver parenchyma, showing T2 hyper- or hypointensity compared to the surrounding liver tissue. **Results**: Liver lesions were found in 9.5% (85/899) of all cMRI studies; of these, 89% ((76/85) of cases) were unknown at time of cMRI, 96% (82/85) were T2 hyperintense, and 38% (32/85) were larger than 1 cm. The patients with liver lesions were older (29 years vs. 22 years, *p* < 0.0001). There were no sex differences in the incidence of liver lesions or differences in right or left ventricular function (LVEF: 57% vs. 58%, *p* = 0.78; RVEF: 55% vs. 54%, *p* = 0.35). The patients with univentricular hearts, transposition of great arteries after atrial switch operation, and atrial septal defects showed the highest incidence (18%, 17%, and 21%, respectively). However, 9% of patients with left heart-sided valve disease also showed liver lesions. **Conclusions**: Incidental findings of liver lesions in cMRI examinations of patients with CHD are reasonably high with almost 10%. In the growing population of adults with CHD, liver monitoring might be helpful to assure overall patient health.

## 1. Introduction

With an incidence of 81 per 10,000 births, congenital heart diseases (CHDs) represent the most common congenital defects [1]. Thanks to advances in treatment strategies, life expectancy of patients with CHD is constantly increasing, leading to an ever-growing new patient population of adults with CHD with previously unknown incidental findings and co-morbidities. One of such findings are liver lesions [2,3]. Benign changes in the liver include hepatocellular adenoma, focal nodular hyperplasia, nodular regenerative hyperplasia, cysts, and hemangiomas. In contrast, malignant liver lesions mainly consist of primary hepatic malignancies, such as hepatocellular carcinoma, as well as secondary metastatic tumors [4]. These lesions can be diagnosed and classified using non-invasive imaging techniques such as sonography or magnetic resonance imaging. Sometimes, a biopsy may be needed to confirm the diagnosis [4]. Different factors might lead to liver parenchymal damage predisposing the development of benign or malign liver lesions. Patients with congenital heart disease frequently exhibit cyanosis or impaired cardiac function with consecutive cellular hypoxia, venous congestion, and low cardiac output, potentially leading to liver remodeling, fibrosis, and cirrhosis [2,5]. In particular. The patients with univentricular hearts and Fontan palliation are known to be at risk for developing liver damage and lesions due to chronic liver congestion [6]. Sugimoto et al. [7] found that approximately 72% to 100% of all Fontan patients suffer from liver disease, however, often asymptomatic [7,8]. Other CHDs are also associated with venous congestion, for example the transposition of the great arteries after atrial switch surgery with systemic right ventricles. Another condition typical of several congenital heart defects leading to liver damage is hypoxia [2,5].

In the present study we aimed to describe the incidence of incidental findings of liver lesions in patients with different congenital heart diseases and look for associations to age, sex, cardiac size, and function. This might help to gain insights into the burden of disease and to potentially identify patients at elevated risk of developing liver lesions.

## 2. Materials and Methods

### 2.1. Patient Cohort

The study was conducted in accordance with the Declaration of Helsinki, and it was approved by the Ethics Committee (protocol code: 266/2024802; 26 June 2024). The patients with congenital heart diseases undergoing cardiac magnetic resonance imaging (cMRI) at Radiology Department between 1st of January 2014 and 10th of April 2024 were reviewed. Using the “Centricity PACS—Radiology RA1000 Workstation and Examination Manager” system, image files were analyzed. Only patients with congenital heart defects (CHDs) were included. The diagnoses were verified using PACS, Centricity Cardio-Workflow V6.0 SP4.4.2, and SAP Healthcare systems. Liver lesions were evaluated using ECG-triggered “Half-Fourier Acquisition Single-Shot Turbo-Spin Echo (HASTE)” sequences performed under breath-hold where feasible with various MRI devices. In most examinations, the superior hepatic segments adjacent to the diaphragm were included in the field of view of the HASTE sequences. The data, including gender, age, CHD type, ventricular function, and liver lesion details (intensity, size, and singularity) were anonymized and recorded in Microsoft Excel. CHDs were categorized into three groups: (1) venous congestion and/or hypoxia group, (2) left ventricular pressure or volume overload group in biventricular patients and (3) low risk group with negligible cardiac burden. Group 1 included patients with univentricular hearts (post-Fontan or Glenn surgery), transposition of the great arteries (TGA), post-atrial switch, arterial switch, or complex TGA, Ebstein anomaly, tetralogy of fallot, pulmonary vein malformation, and atrial septal defect (ASD) (isolated or combined with other CHDs). Group 2 included patients with other valve disease (mainly aortic or mitral valve disease), interrupted aortic arch and LV volume load congenital shunts (ventricular septal defect, persistent ductus arteriosus botalli). Group 3 included patients with isolated right aortic arch, dextrocardia, or congenital aortic aneurysm. The patient cohorts are presented in Table 1. Generative artificial intelligence (OpenAl, ChatGPT, GPT-5.3) was used for language editing and refinement of the manuscript. The tool did not contribute to data collection, analysis, or interpretation.

### 2.2. Ventricular Function and Size

All data on ventricular size and function were taken from the medical reports. These data were originally measured using transversal cardiac cine images. The LVEF measurements were available for 885/899 patients (98%), and RVEF measurements were made available for 854/899 patients (95%). The LVEDVi data were available for 816/899 patients (91%), whereas the RVEDVi data were available for 803/899 patients (89%). Indexed left and right ventricular end-diastolic volumes (LVEDVi and RVEDVi) for the total cohort showed mean values of 87 ± 22 mL/m^2^ and 99 ± 33 mL/m^2^, respectively (Table 2).

### 2.3. Data Analysis

A total of 899 cardiac MRI scans were reviewed. Liver lesions were defined as round structures with distinct morphologies differing from normal liver parenchyma or vessels. Presence, amount, size, and T2 intensity of liver lesions were determined in T2-weighted images using the PACS system. All cardiac MRI scans were reviewed, and liver lesions were marked and measured by a medical student. Validation was performed and final conclusions reached consensus based on opinions formed by medical experts (a radiologist, pediatrician, and pediatric cardiologist).

The data regarding demographic information, medical history, and ventricular size and function were taken from the archived medical reports. The data were collected, and mean values, standard deviations, variances, and medians were calculated and graphically represented. Gender-specific differences in liver lesions were analyzed using the Fisher exact test in Jamovi 2.5 [9]. Further statistical analysis, including the Mann–Whitney-U test, was performed in Excel using the XLSTAT add-on. Age and EF were preliminarily assessed for normal distribution with Q-Q plots before further analysis, with *p*-values calculated to determine result significance.

## 3. Results

### 3.1. Incidental Findings of Hepatic Abnormalities in Patients with Congenital Heart Disease

**Figure 1:**
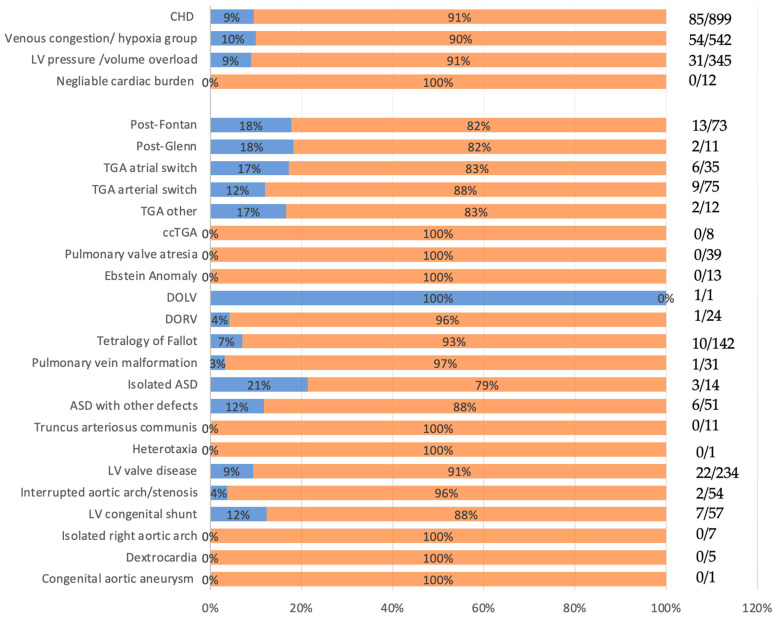
The comparison of the number of patients with (orange) and without (blue) liver lesions; ASD: atrial septal defect, ccTGA: congenital corrected transposition of the great arteries, CHD: congenital heart disease, DOLV: double outlet left ventricle, DORV: double outlet right ventricle, LV: left ventricle, TAC: truncus arteriosus communis, TGA: transposition of the great arteries, TOF: tetralogy of fallot.

Liver lesions were found in 85/899 (9.5%) patients with congenital heart defects (Figure 1). 

**Figure 2:**
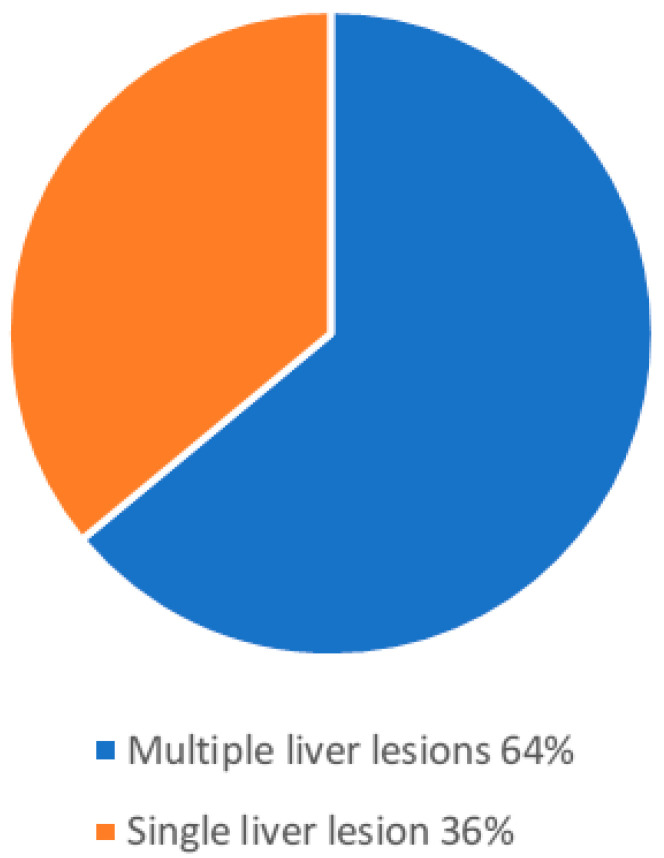
Number of liver lesions per patient with congenital heart disease, categorized as one liver lesion or more than one liver lesion.

A total of 53/85 (62%) liver lesions were less than or equal to 1 centimeter in diameter. A total of 32/85 (38%) liver lesions were larger than one centimeter. It was observed that some patients exhibited multiple, albeit often small, lesions in the liver (Figure 2). 

**Figure 3:**
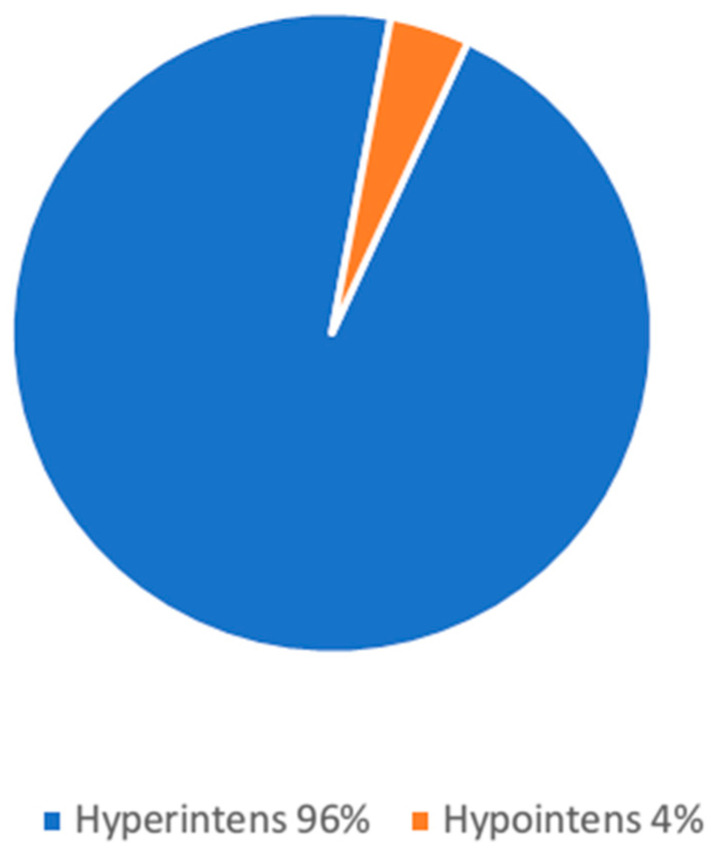
Distribution of liver lesion intensity on T2-weighted images.

**Figure 4:**
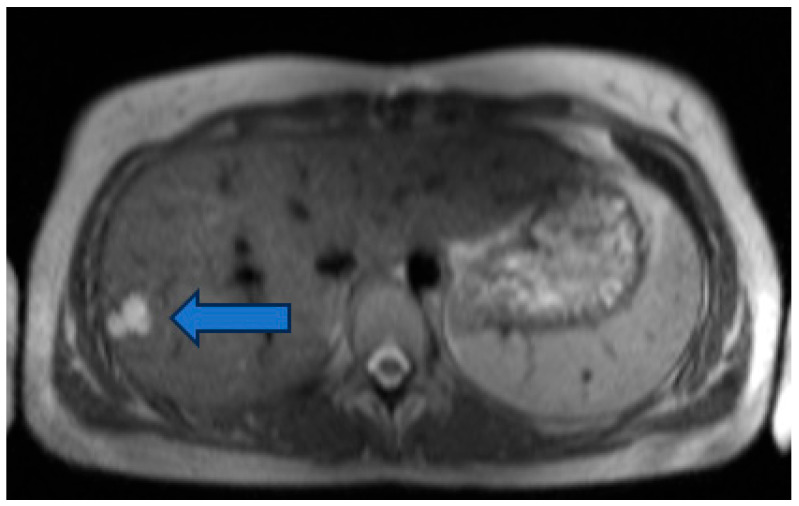
T2-hyperintense liver lesion (indicated by the blue arrow) in HASTE sequence in a patient with double outlet left ventricle.

**Figure 5:**
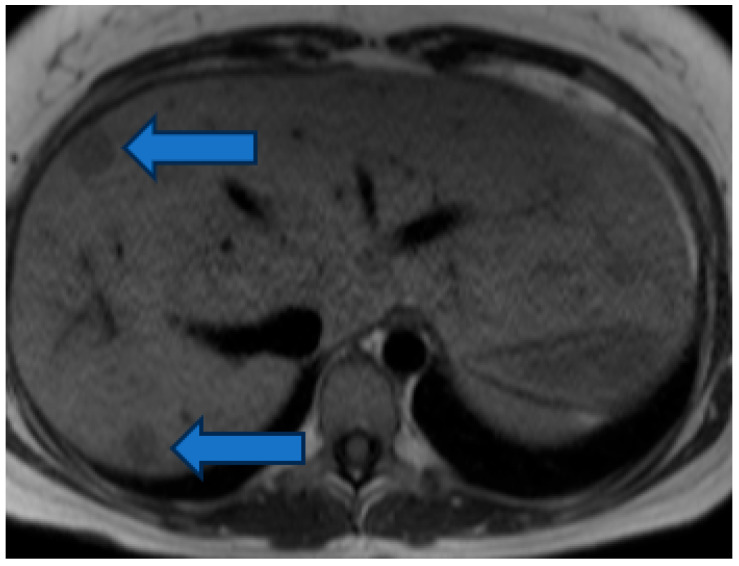
T2-hypointense liver lesions (indicated by the blue arrows) in HASTE sequence in a patient with atrial septal defect.

A total of 54/85 (64%) patients with liver lesions had a single liver lesion, and 31/85 (36%) patients more than one liver lesion. A total of 96% of the liver lesions showed hyperintense signal in the T2 HASTE sequence, and 4% were classified as hypointense (Figure 3, Figure 4 and Figure 5). 

**Figure 6:**
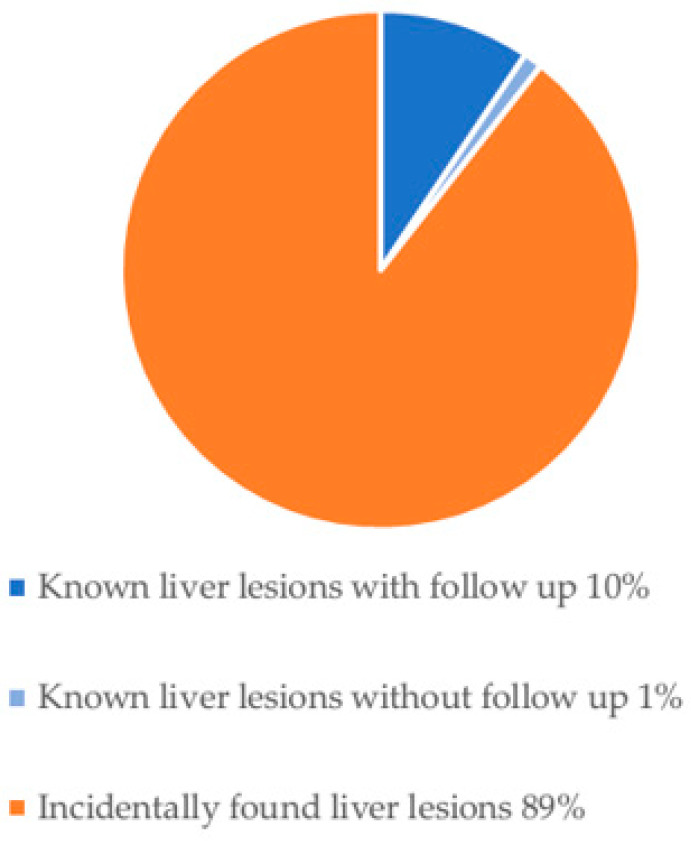
Breakdown of patients with liver lesion into already diagnosed liver lesions with or without follow-up examination and incidentally found liver lesions.

**Figure 7:**
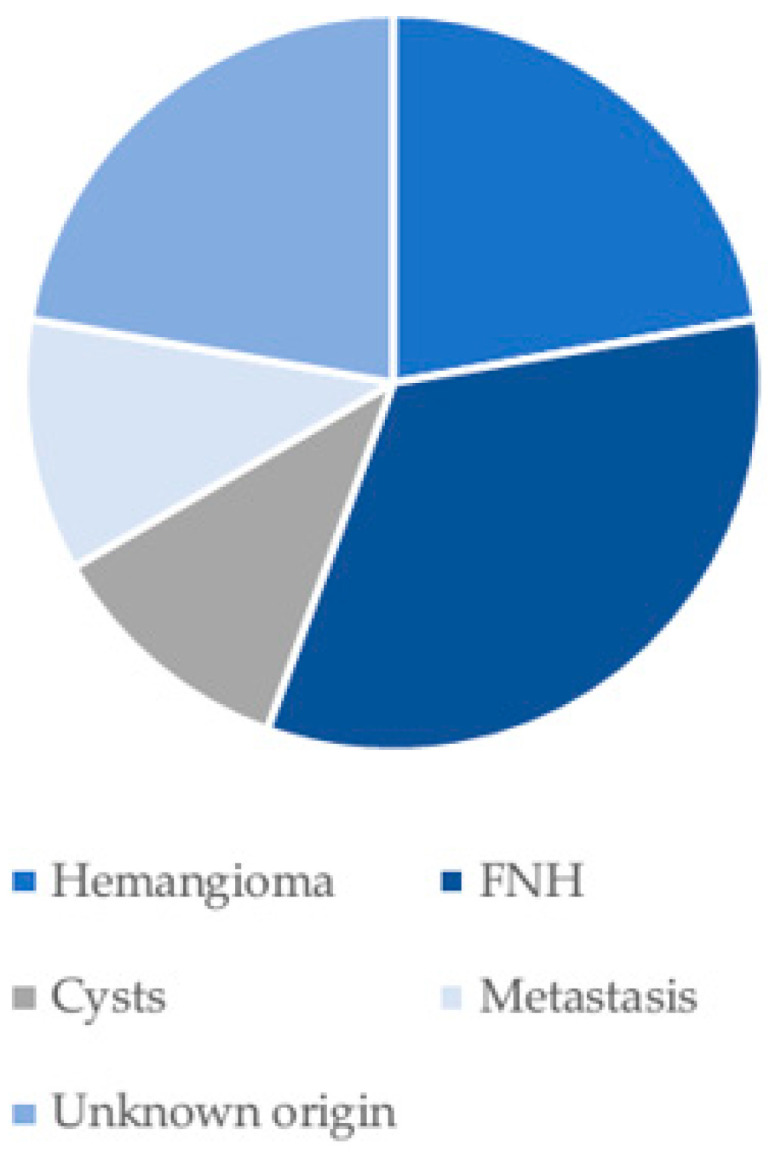
The diagnosis of the already known liver lesions; FNH: focal nodular hyperplasia.

Hypointense liver lesions were identified in one patient with a univentricular heart and Fontan surgery, in one patient with atrial septal defect in conjunction with another congenital heart defect (aorta ascendens ectasia, pulmonary stenosis, sinus venosus defect, and interrupted aortic arch), and one patient with ventricular septal defect. Liver lesions were already described before in nine out of 85 (11%) patients (focal nodular hyperplasia (FNH) in three patients, hemangioma in two patients, cyst in one patient, metastasis in one patient and two of unknown origin). In 76 out of 85 (89%) patients, no diagnosis of a liver lesion was described before (Figure 6 and Figure 7).

### 3.2. Association of Hepatic Findings with Age, Sex, Cardiac Size, and Function in Patients with Congenital Heart Disease

#### 3.2.1. Age Difference Between Patients with and Without Liver Lesions

The patients with liver lesions were significantly older than patients without liver lesions, looking at the total cohort, the venous congestion/hypoxia group, and the LV pressure/volume overload group (Table 3). In patients in the negligible cardiac burden group, no liver lesions were found, and no such statistic could be performed; however, these patients were also younger than patients in the two other groups.

#### 3.2.2. No Sex Differences Were Found in the Incidence of Hepatic Lesions in Patients with Congenital Heart Disease

In our population, there were 348 women with CHD, of which 35 (10%) showed liver lesions, and 551 men, of which 50 (9%) showed liver lesions. The incidental finding of liver lesions was not different between females (35/348) and males (50/551) (*p* = 0.5). This was also seen in the subgroup of patients with venous congestion/hypoxia (*p* = 0.9), in patients with left ventricular pressure or volume load (*p* = 0.3), and any other subgroup presented in this manuscript.

#### 3.2.3. Patients with Congenital Heart Disease and Liver Lesions Did Not Show Differences in Left or Right Ventricular Size and Function Compared to Patients Without Liver Lesions

In the total patient cohort, the patients with liver lesions showed similar left and right ventricular ejection fraction (LVEF, RVEF) compared to patients without liver lesions (mean LVEF 57% (±9%) vs. 58% (±9%), *p* = 0.8) (mean RVEF 55% (±10%) vs. 54% (±10%) (*p* = 0.4)).

Similar results were seen in the subgroups: the patients from the venous congestion/hypoxia group showed (mean LVEF 55% (±10%) vs. 56% (±9%) (*p* = 0.7)) (mean RVEF 53% (±10%) vs. 51% (±11%) (*p* = 0.1)); and the patients with LV pressure/volume load showed (mean LVEF is 60% (±8%) vs. 60% (±8%) (*p* = 0.9) (mean RVEF 57% (±9%) vs. 58% (±8%) (*p* = 0.7)). As no liver lesions were observed in patients with negligible cardiac burden, subgroup comparison was not feasible.

LVEF was categorized into preserved EF (pEF) (≥50%), mildly reduced EF (mrEF) (41–49%), and reduced EF (rEF) (≤40%). Overall, the patient cohort with liver lesions can be classified into 67 patients (85%) with pEF, eight patients (10%) with mrEF, and four patients (5%) with rEF. Of all patients without liver lesions, 638 (84%) could be categorized as pEF, 86 patients (11%) as mrEF, and 24 patients (3%) as rEF. The remaining patients had to be excluded due to missing data.

Based on guidelines by Lang et al. [10], RVEF was categorized as preserved (≥45%) or reduced (<45%). Overall, in the patient cohort with liver lesions, 54 (69%) had an RVEF above 45%, and 20 (26%) had an RVEF below 45%. Of all patients without liver lesions, 635 (82%) had an RVEF above 45%, and 101 (13%) had an RVEF below 45%. In the cohort with liver lesions, five patients (5%) had to be excluded due to missing data, as well as 39 patients (5%) in the cohort without liver lesions. Using a chi-square test, it was determined that the patients with liver lesions showed a RVEF below 45% significantly more often than patients without liver lesions (*p* = 0.02).

End-diastolic volumes of left and right ventricles did not differ between patients with and without liver lesions (mean LVEDVi 88 mL/m^2^ (±22 mL/m^2^) vs. 86 mL/m^2^ (±23 mL/m^2^) (*p* = 0.7)) (mean RVEDVi 96 mL/m^2^ (±36 mL/m^2^) vs. 102 mL/m^2^ (±31 mL/m^2^) (*p* = 0.06)). Furthermore, no significant differences could be observed in all CHD subgroups.

## 4. Discussion

### 4.1. Key Findings

In the present retrospective study, we found liver lesions in 9.5% of 899 patients with congenital heart defects undergoing cMRI at our clinic within the last 10 years. The majority of lesions were incidental new findings. The rate was the highest in the patients with univentricular hearts post-Glenn and post-Fontan surgery or in patients with transposition of the great arteries, for example. Most liver lesions were solitary, smaller than 1 cm in size, and hyperintense on T2-weighted HASTE sequences. Given the small size of most lesions (<1 cm) and the limited liver coverage of cardiac MRI, their clinical significance remains uncertain. In clinical practice, such incidental findings are typically further evaluated only when imaging characteristics are indeterminate or when lesions exceed approximately 1 cm in size, usually by targeted liver imaging such as abdominal ultrasound or dedicated liver MRI. The patients with liver lesions were older than those without, which was consistent across all subgroups with right and left heart burden. There were no sex differences and no significant differences in left or right cardiac size. The patients with liver lesions more often presented with reduced right ventricular function; however there was no difference in mean right or left ventricular function between patients with and without liver lesions.

### 4.2. Comparison with Similar Research and Explanation of Findings

Prior findings by Reiter et al. [2,3] describe liver damage as an important topic in congenital heart diseases, and it is more common in patients with univentricular hearts post-Fontan surgery, as well as in patients with TGA after atrial switch and patients with Eisenmenger syndrome [11]. Consistent with that, we also found the highest rates of liver lesions in patients with univentricular heart disease and TGA patients. On the other side, there was also a high rate of liver lesions in patients with an atrial septal defect, which is hemodynamically much less significant compared to univentricular hearts, and in patients with left-sided valve diseases. The relatively high incidence observed in ASD patients should be interpreted with caution. Isolated ASD rarely leads to chronic hepatic congestion, and the small number of patients in this subgroup may have influenced the observed rate. In our total cohort, liver lesions were observed at comparable frequencies in patients with right- and left-sided cardiac burden, suggesting that factors beyond venous congestion alone may contribute to their development [2,3].

In general, benign liver lesions are known to have a higher incidence rate in older patients and in women. Age may represent an important confounding factor, as benign liver lesions are known to increase with age in the general population. The higher age observed in patients with liver lesions in our cohort may therefore partly explain the detected incidence. Hemangiomas, for example, are reported to have an incidence of up to 20% in patients between 30 and 50, with a significantly higher rate in women [12]. In our study, patients with CHD were mostly younger than 30, but we still found that the patients with liver lesions were significantly older than the patients without liver lesions. While these findings may indicate an age-related development of liver lesions independent of congenital heart disease, it is also conceivable that long-standing cardiac conditions could facilitate earlier manifestation compared with the general population; however, this cannot be directly assessed within the present study [13]. As no control group from the general population was included, it cannot be determined whether the observed liver lesions are specifically associated with congenital heart disease or if they represent incidental findings.

In the Fontan patients, for example, we found liver lesions in 18% of cases. Other studies even described an incidence of focal liver lesions of 69% in Fontan patients with a mean age of 20 years; however, in a cohort undergoing specialized liver MRI, liver abnormalities were already expected [13]. Consistent with Diaz et al. [13], who did not find sex differences in the presence of liver lesions in Fontan patients, no significant sex-specific differences were observed across the overall patient population and within the different subgroups.

According to Karmazyn et al., liver lesions larger than 5 cm may warrant histological evaluation, whereas smaller lesions can be classified into typical benign lesions (e.g., cysts, hemangiomas, and focal nodular hyperplasia) and atypical lesions requiring closer assessment or follow-up imaging. In our cohort, the majority of liver lesions demonstrated imaging characteristics consistent with benign entities looking at their signal characteristics and morphological appearance. This observation is in line with previous reports describing benign-appearing liver lesions in the context of congenital heart disease and venous congestion [14,15]. However, since liver lesions were only detected in T2-weighted images only partly covering the liver and the absence of specialized liver protocol, the final characterization of the incidentally detected liver lesions cannot be made.

In our study cohort, we did not find significant differences in mean right and left ventricular function or in end-diastolic volumes between patients with and without liver lesions. It might be possible that ventricular stiffness and decreased diastolic function might be associated with the presence of liver lesions; however, these parameters were not available in the present study. Innocenzi et al. described an elevated EDVi in Fontan patients and a subsequent positive correlation with liver stiffness [16]. However, liver stiffness has been shown to be negatively associated with liver lesions in Fontan patients according to Diaz et al. [13].

### 4.3. Interpretation of Unknown Liver Lesions

An important consideration in this study is the relatively high incidence of previously undetected liver lesions in patients with congenital heart disease. Since the longitudinal course of these findings is unknown, our study emphasizes the necessity of a systematic review of incidental liver lesions in patients with congenital heart disease.

The unknown liver lesions in this cohort could reflect a variety of potential etiologies, with venous congestion being a likely contributor. Patients with univentricular heart physiology, particularly those who have undergone Fontan or Glenn surgeries, are at a higher risk for chronic venous congestion, which can lead to hepatic fibrosis or cirrhosis over time [17]. The chronic congestion, impaired venous return, and altered blood flow seen in these patients are well-established factors in liver pathology and could be predisposing factors for the development of focal liver lesions.

Many of the smaller lesions could correspond to benign cysts or insignificant findings that do not warrant additional diagnostics. Conditions such as hepatocellular adenomas, regenerative nodules, or even nodular regenerative hyperplasia (NRH) are often associated with altered liver circulation, and these might present as small hyperintense areas on T2-weighted MRI images [4,15]. These lesions may not show up on standard imaging but could still be clinically significant, especially in patients with chronic heart failure or cyanosis, conditions frequently seen in congenital heart disease [2,18].

Additionally, these hyperintense lesions might reflect early hepatic steatosis (fatty liver) or early fibrosis that does not yet show the more distinctive characteristics of established liver disease. Such lesions could be indicative of subclinical liver changes due to chronic venous congestion, hypoxia, or other pathophysiological processes inherent to congenital heart disease. In fact, the liver involvement in CHDs may be underappreciated, as some of these changes might not be evident clinically or on conventional imaging [19].

Though less likely in this age group, the possibility of metastatic lesions should be ruled out, particularly in older patients or those with known malignancies. Other rare causes, such as hematomas, abscesses, or lesions linked to systemic circulatory changes, could also account for some of the unknown lesions, although these are less common in this cohort [2,20]. The identification and the subsequent clarification of their significance represent an important consideration for both clinical practice and further research.

In many cases, small incidental liver lesions detected on non-dedicated imaging remain clinically insignificant and do not require immediate changes in patient management. However, indeterminate findings or lesions with atypical imaging characteristics usually warrant further targeted liver imaging and clinical follow-up.

### 4.4. Implications and Actions Needed

The high incidence of incidental hepatic findings observed in our cohort highlights the need for structured diagnostic and follow-up strategies in patients with congenital heart disease. At our institution, incidental liver abnormalities detected on cardiac MRI are reviewed jointly by cardiologists and radiologists. If a focal liver lesion cannot be clearly characterized on the cardiac MRI sequences, further evaluation is recommended. This diagnostic approach was also applied to the patients included in our cohort.

In most cases, the next diagnostic step consists of targeted liver imaging, typically abdominal ultrasound or dedicated liver MRI with contrast enhancement, depending on lesion characteristics and patient history. Findings suspected to represent benign entities such as cysts or hemangiomas are usually documented and followed conservatively. Indeterminate lesions are further evaluated through specialized liver imaging and, if necessary, hepatology consultation. Patients with confirmed or suspected hepatic pathology are integrated into a structured follow-up program. Follow-up intervals depend on lesion characteristics and underlying risk factors, including long-standing Fontan circulation or hepatic congestion. These measures aim to ensure early detection of clinically relevant liver disease while avoiding unnecessary diagnostic procedures [4,12].

### 4.5. Limitations and Strengths

Limitations of this study include its single center, retrospective design and the lack of liver-specific magnetic resonance imaging sequences. For liver lesion detection, HASTE sequences of the cardiac MRI protocol were used, which have not always covered the entire liver, leading to a possible underestimation of the rate of liver lesions. Another limitation is that only patients with congenital heart diseases and indication for a cardiac MRI were included; thus, findings of the incidence of liver lesions cannot be transferred to all patients with CHDs. Patients with congenital heart disease who do not undergo cardiac MRI may have different clinical characteristics.

However, it is noteworthy that our institution is a specialized center for the treatment of congenital heart defects, and a considerable number of patients (*n* = 899) could be included into the study.

## 5. Conclusions

The study demonstrated that liver lesions are not uncommon in patients with congenital heart defects undergoing cardiac MRI examinations. It is particularly noteworthy that liver lesions were observed not only in patients with heart defects accompanied by significant hemodynamic alterations, such as the Fontan circulation, but also in those with less pronounced hemodynamic changes. The patients with liver lesions were older than patients without liver lesions; however, no association with sex or cardiac size and function could be found. The results of this study are a first insight into the incidence of liver lesions in patients with different congenital heart diseases, and they suggest that especially young adults with congenital heart disease would benefit from periodic liver screening and subsequent regular follow-up examinations throughout their lives.

## Figures and Tables

**Table 1:** Patient cohort with age, sex distribution, and ventricular type.

CHD Type	Number (%)	Mean Age (SD)	Sex, Female (%)	Patient Group
Total cohort	899 (100%)	23 (±12)	39%	1,2,3
Venous congestion/hypoxia group	542/899 (60%)	23 (±12)	39%	1
LV pressure/volume overload	345/899 (39%)	23 (±12)	37%	2
Negligible cardiac burden	12/899 (1%)	17 (±12)	50%	3
**Specific Conditions**				
**Univentricular heart** Post-Fontan	73/899 (4%)	20 (±9)	41%	1
Post-Glenn	11/899 (1%)	18 (±13)	36%	1
TGA (Post-atrial switch)	35/899 (4%)	34 (±8)	29%	1
TGA (Post-arterial switch)	75/899 (8%)	21 (±6)	34%	1
TGA (Other surgeries)	12/899 (1%)	22 (±12)	33%	1
ccTGA	8/899 (1%)	26 (±15)	38%	1
Pulmonary valve atresia	39/899 (4%)	28 (±11)	41%	1
Ebstein anomaly	13/899 (1%)	22 (±14)	23%	1
DOLV	1/899 (0.11%)	23 (±0)	100%	
DORV	24/899 (3%)	33 (±13)	54%	
Tetralogy of Fallot	142/899 (16%)	20 (±12)	37%	1
Pulmonary vein malformation	31/899 (3%)	28 (±15)	39%	1
Isolated ASD	14/899 (2%)	24 (±22)	43%	1
ASD with other defects	51/899 (6%)	16 (±11)	59%	1
Truncus arteriosus communis	11/899 (1%)	21 (±6)	45%	1
Heterotaxy	1/899 (0.11%)	22 (±0)	100%	1
LV valve disease	234/899 (26%)	23 (±11)	35%	2
Interrupted aortic arch	54/899 (6%)	21 (±15)	37%	2
LV congenital shunt	57/899 (6%)	22 (±10)	46%	2
Isolated right aortic arch	7/899 (0.8%)	23 (±13)	57%	3
Dextrocardia	5/899 (0.6%)	12 (±5)	40%	3
Congenital aortic aneurysm	1/899 (0.11%)	0	0%	3

ASD: atrial septal defect, ccTGA: congenital corrected transposition of the great arteries, CHD: congenital heart disease, DOLV: double outlet left ventricle, DORV: double outlet right ventricle, LV: left ventricle, TAC: truncus arteriosus communis, TGA: transposition of the great arteries, TOF: tetralogy of fallot.

**Table 2:** Mean, standard deviation, variance, and median of left and right ventricular ejection fraction and indexed end-diastolic volume; 14 patients had to be excluded due to missing data.

CHD	Mean LVEF (±SD)	Mean RVEF (±SD)
Total cohort	58% (±9)	54% (±10)
Venous congestion/hypoxia group	56% (±9)	51% (±11)
LV pressure/volume overload	60% (±8)	58% (±8)
Negligible cardiac burden	62% (±6)	62% (±7)
**Specific Conditions**		
Post-Fontan operation	50% (±9)	47% (±8)
Post-Glenn operation	49% (±11)	39% (±13)
TGA (Post-atrial switch)	57% (±11)	45% (±12)
TGA (Post-arterial switch)	56% (±7)	57% (±8)
TGA (Other surgeries)	54% (±9)	41% (±8)
ccTGA	59% (±11)	51% (±12)
Pulmonary valve atresia	52% (±10)	46% (±11)
Ebstein anomaly	56% (±4)	41% (±16)
DOLV	47% (±0)	38% (±0)
DORV	55% (±7)	52% (±8)
Tetralogy of Fallot	57% (±8)	50% (±9)
Pulmonary vein malformation	61% (±6)	57% (±7)
Isolated ASD	58% (±7)	54% (±10)
ASD with other defects	54% (±13)	55% (±10)
Truncus arteriosus communis	52% (±10)	51% (±9)
Heterotaxy	60% (±0)	65% (±0)
LV valve disease	60 (±8)	58% (±8)
Interrupted aortic arch/stenosis	61 (±8)	59 (±7)
LV congenital shunt	59 (±10)	59 (±8)
Isolated right aortic arch	65% (±4)	63% (±8)
Dextrocardia	58% (±6)	60% (±4)
Congenital aortic aneurysm	N/A	51%

ASD: atrial septal defect, ccTGA- congenital corrected transposition of the great arteries, CHD: congenital heart disease, DOLV: double outlet left ventricle, DORV: double outlet right ventricle, LV: left ventricle, TGA: transposition of the great arteries.

**Table 3:** Age distribution and significant difference between patients with and without hepatic findings; LV: left ventricular, N/A: not applicable, SD: standard deviation.

Patient Group	With Hepatic Finding Mean (±SD)	Without Hepatic Finding Mean (±SD)	*p*-Value
Total cohort	29 years (±15)	22 years (±11)	<0.0001
Venous congestion/hypoxia group	35 years (±19)	22 years (±11)	0.011
LV pressure/volume overload	29 years (±7)	23 years (±12)	<0.001
Negligible Cardiac Burden	N/A	17 years (±12)	N/A

## Data Availability

The data presented in this study are available on request from the corresponding author due to privacy and ethical restrictions related to patient data.

## Abbreviations

The following abbreviations are used in this manuscript:

ASD	atrial septal defect
ccTGA	congenital corrected transposition of the great arteries
CHD	congenital heart disease
cMRI	cardiac magnetic resonance imaging
DOLV	double outlet left ventricle
DORV	double outlet right ventricle
EDVi	indexed end-diastolic volume
FNH	focal nodular hyperplasia
HASTE	Half-Fourier Acquisition Single-Shot Turbo Spin Echo
LV	left ventricle
LVEDVi	indexed left ventricular end-diastolic volume
LVEF	left ventricular ejection fraction
mrEF	mildly reduced ejection fraction
MRI	magnetic resonance tomography
N/A	not applicable
OP	operation—surgery
pEF	preserved ejection fraction
rEF	reduced ejection fraction
RV	right ventricle
RVEDVi	indexed right ventricular end-diastolic volume
RVEF	right ventricular ejection fraction
SD	standard deviation
SV	systemic ventricle
TAC	truncus arteriosus communis
TGA	transposition of the great arteries
TOF	tetralogy of Fallot
